# Videogame interventions and spatial ability interactions

**DOI:** 10.3389/fnhum.2014.00183

**Published:** 2014-03-26

**Authors:** Thomas S. Redick, Sean B. Webster

**Affiliations:** Department of Psychological Sciences, Purdue UniversityWest Lafayette, IN, USA

**Keywords:** video games, training, transfer, intelligence, cognitive interventions

## Abstract

Numerous research studies have been conducted on the use of videogames as tools to improve one’s cognitive abilities. While meta-analyses and qualitative reviews have provided evidence that some aspects of cognition such as spatial imagery are modified after exposure to videogames, other evidence has shown that matrix reasoning measures of fluid intelligence do not show evidence of transfer from videogame training. In the current work, we investigate the available evidence for transfer specifically to nonverbal intelligence and spatial ability measures, given recent research that these abilities may be most sensitive to training on cognitive and working memory tasks. Accordingly, we highlight a few studies that on the surface provide evidence for transfer to spatial abilities, but a closer look at the pattern of data does not reveal a clean interpretation of the results. We discuss the implications of these results in relation to research design and statistical analysis practices.

## Videogame interventions and spatial ability interactions

In the past 10 years, there has been substantial interest in the idea that playing videogames may serve to improve certain cognitive functions. A recent meta-analysis (Powers et al., 2013) provided a quantitative summary of the numerous studies in which a videogame-playing group was compared against a control group that did not receive the videogame “treatment” of interest. As is typically done in meta-analyses, Powers et al. (2013) used broad operational definitions of cognitive outcomes, such as combining together various outcome measures of “executive functions”, which included multitasking, inhibition, task-switching, short-term/working memory, and intelligence. Overall, videogame training effects on executive functions was statistically significant (*d* = 0.16), although the effect would be classified as small according to Cohen (1992). Notably, transfer to intelligence was not significant, *d* = 0.06, and inhibition was the only executive function that was significantly improved by videogame training. On the other hand, tests of spatial imagery, such as mental rotation tasks, exhibited stronger meta-analytic effects, *d* = 0.43. Our current work investigates specifically the effect of videogame training on spatial ability transfer outcomes, given recent research that argues these spatial ability tests may be most sensitive to cognitive training (Colom et al., 2013).

**Figure 1:**
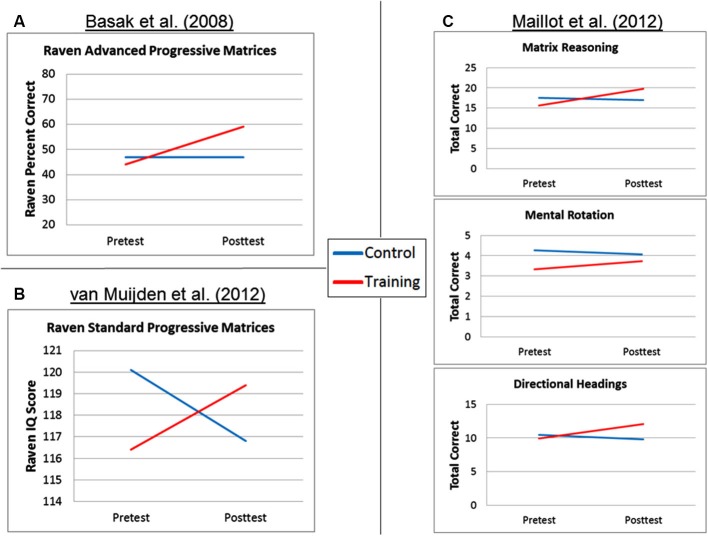
**Spatial ability transfer results as a function of group for (A) Basak et al. (2008); (B) van Muijden et al. (2012); and (C) Maillot et al. (2012)**.

Basak et al. (2008) provide an illustrative example of almost ideal intelligence transfer results, as a function of videogame training. In their study, older adults in the training group played a videogame (*Rise of Nations*) during 15 sessions. Raven Advanced Progressive Matrices, a matrix reasoning test commonly used to measure fluid or nonverbal intelligence, was among the battery of tests administered during pre-test and post-test transfer sessions. As seen in Figure 1A, the training group improved on Raven scores from pre- to post-test, whereas the control group that did not do anything between pre- and post-test (a *no-contact control group*) showed no improvement on Raven. In addition, the Raven mean pre-test scores were similar for the two groups. The interaction pattern in Basak et al. (2008) is straightforward to interpret, and the transfer data easily support the argument that the training “worked” for those subjects.

The pattern of transfer results observed in Basak et al. (2008) is what one would predict if the pre- to post-test change for the training group is what drives the observed significant interaction. However, videogame training studies such as Maillot et al. (2012), van Muijden et al. (2012), and Cherney (2008) are more difficult to interpret, because the pattern of transfer results is not similar to the Basak et al. (2008) example above. As outlined in the summaries below, each of these studies has been used to provide support for the efficacy of videogame interventions upon spatial abilities, yet a closer examination of the results warrants a more cautious interpretation. Note that our discussion in the current manuscript focuses on interactions and how strongly one can interpret the results as support for the efficacy of videogame interventions; recent articles have discussed many other methodological and measurement issues in videogame and brain training studies (e.g., Boot et al., 2011, 2013; Shipstead et al., 2012; Green et al., 2013).

## Van Muijden et al. (2012)

Two groups of participants completed the study: the video game group (*n* = 53) and the documentary group (*n* = 19). Participants in the videogame training group played five custom-built games (cf. Figure 1; van Muijden et al., 2012). Participants in the documentary control group watched documentaries of about 30 min in length followed by a three-to-five multiple-choice question quiz about the documentary. Participants in both the videogame and documentary control groups were instructed to complete one 30-min session daily for 7 weeks, and participants that completed the study completed on average over 21 h of the prescribed activity. Before and after the videogame and documentary sessions, all participants completed a cognitive test battery that consisted of nine cognitive tests, including Raven standard progressive matrices. Raw scores from Raven were converted to IQ scores. van Muijden et al. (2012) concluded that “the results from the present study suggest that modest improvements of inductive reasoning can also be achieved by means of playing cognitive training games” (p. 10).

The group × session interaction on Raven was significant ($\eta_{p}^{2}=0.068$), and is displayed in Figure 1B. However, this pattern is quite different from the Raven interaction pattern shown by Basak et al. (2008). The pre-test Raven IQ mean score for the videogame group was 116.4 and the post-test mean score was 119.4, an increase of 3.0 IQ points. The pre-test Raven IQ mean score for the documentary group was 120.1 and the post-test mean score was 116.8, a decrease of 3.3 IQ points. Although the between-groups comparison of the pre-test scores reported by van Muijden et al. (2012) was not significant (“*p* > 0.05”, p. 4), the effect size for the pre-test scores indicated a small-to-medium difference (Cohen’s *d* = 0.38). van Muijden et al. (2012) conducted subsequent simple main effect analyses to examine the pre- to post-test change within each group separately. Within the videogame group only, the pre- to post-test Raven increase was considered marginally significant (*p* = 0.05), whereas the pre- to post-test Raven decrease for the documentary group was not significant (*p* > 0.10). Critically, however, there was also a large difference in the group sample sizes (*n* = 53 and *n* = 19 for the videogame training group and the documentary control group, respectively). The sample size difference is extremely important as it pertains to the aforementioned follow-up tests—the reported effect size for the pre- to post-test Raven change score was actually larger for the nonsignificant control group ($\eta_{p}^{2}=0.068$) than it was for the significant training group ($\eta_{p}^{2}=0.071$). Thus, despite the significant interaction and associated effect size, we view the crossover pattern of Raven results as relatively weak evidence for the efficacy of videogame training to improve spatial abilities.

## Maillot et al. (2012)

Two groups of older adults (between the ages of 65 and 78 years old) completed the study: one group (*n* = 15) was assigned to the exergame training condition and the other (*n* = 15) was assigned to the no-training control group. The exergame training group completed two 1-h exergame sessions per week for 12 weeks for a total training time of 24 h. During these sessions participants played *Wii Sports*, *Wii Fit*, and *Mario and Sonic on Olympic Games*. In pre- and post-test sessions, all participants completed a battery of cognitive assessment tasks—we focus here on the matrix reasoning test (a subtest of the Wechsler Abbreviated Scale of Intelligence), a mental rotation task, and a directional headings test, all measures of spatial ability. Maillot et al. (2012) concluded “exergame training, which combines cognitive and physical demands in an intrinsically attractive activity, might be an effective way to promote physical and cognitive improvements among older adults” (p. 597).

In the published article, only the pre-test/post-test change scores were reported for each test, but the pre- and post-test values were provided upon request (P. Maillot, personal communication, 10/9/13), and are shown in Figure 1C. First, Maillot et al. (2012) reported that the mental rotation task change scores were not significantly different between the exergame and control groups (*p* = 0.24, $\eta_{p}^{2}=0.019$), so this result is not discussed further. Maillot et al. (2012) reported that the matrix reasoning test change scores were significantly different between the exergame and control groups (*p* < 0.01) with a very large effect size of $\eta_{p}^{2}=0.531$. For the directional headings test, Maillot et al. (2012) reported that the change scores were significantly different between the exergame and control groups (*p* = 0.02), again with a large effect size of $\eta_{p}^{2}=0.149$. Critically, across all three dependent variables, the control group shows a numerical decrease from pre-test to post-test on each test, and the control group pre-test scores were numerically larger than the training group. Given the small sample sizes, between-groups comparisons of the pre-test scores were not significant (all *p*’s > 0.13), yet the effect sizes for the pre-test scores indicated small-to-medium differences (Cohen’s *d* = 0.14–0.59). Although the matrix reasoning and directional headings difference scores were significantly different between the training and control groups, examination of the pre- and post-test scores instead of only the change scores reveals an interaction pattern that complicates a strong interpretation of the exergame training efficacy. Combined with the absence of exergame effects on the mental rotation task and the use of a no-contact control group, we view the spatial ability transfer results here as modest at best, which contrasts with the large effect sizes reported in the article.

## Cherney (2008)

There has been substantial interest in the idea that videogames could reduce or eliminate gender effects in spatial ability, following the study by Feng et al. (2007). Cherney (2008) investigated gender differences in videogame effects upon spatial abilities, reported in a paper titled “Mom, let me play more computer games: They improve my mental rotation skills”. Separate groups of male (*n* = 30) and female (*n* = 31) undergraduate students completed the study. Participants were randomly assigned to one of the three conditions: 3D videogame training (*n* = 20), 2D videogame training (*n* = 21), or control (*n* = 20) group, such that there were either 10 or 11 participants of each gender in each group (personal communication, I. Cherney, 11/25/2013). The 3D group played *Antz Extreme Racing*, the 2D group played *Tetrus*, and the remaining control participants played paper-and-pencil logic games. Thirty-six participants completed the training during 2 weeks and 25 participants completed the training during 1 week. The participants all completed mental rotation and card rotation spatial ability tests in pre- and post-test sessions before and after the training period, respectively. Cherney (2008) concluded that “the results suggest that even a very brief practice (4 h) in computer game play does improve performance on mental rotation measures” (p. 783), and specifically, “practice with the Antz game, a 3-D computer game, seemed particularly beneficial for women’s Vandenberg mental rotation test (VMRT) scores” (p. 785).

**Figure 2:**
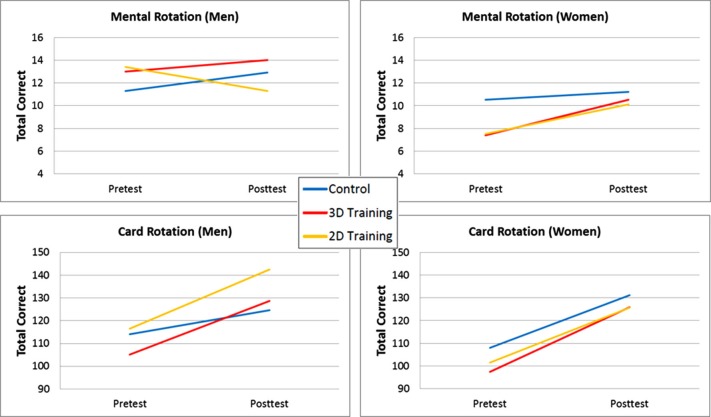
**Spatial ability transfer results as a function of gender and group for Cherney (2008)**.

Figure 2 displays the results for each transfer test as a function of group and gender (mean and standard deviations provided by I. Cherney, personal communication, 10/17/13). Collapsing across training and control groups, Cherney (2008) reported that there were significant improvements on the mental rotation test scores for women (*p* < 0.001) but not for men, and that card rotation test scores improved for both men and women (*p* < 0.001). Unfortunately, the necessary statistical comparisons testing whether the male and female training and control groups differentially improved from pre- to post-test on each dependent variable were not reported in Cherney (2008). As seen in Figure 2, the results are not clear. For the mental rotation test, men playing the 2D Tetrus game showed almost the same amount of decrease in scores from pre- to post-test (2.1 items) that the women increased in scores from pre- to post-test (2.6 items). In addition, for the mental rotation test, the effect of 3D and 2D training for the female groups was to bring their post-test scores up to the level of the pre-test mental rotation score for the female control group. For card rotation, Cherney reported significant pre- to post-test increases for each of the six groups tested, but no comparison of differences in the gain scores across groups. Given the small sample sizes, between-groups comparisons of the mental rotation and card rotation pre-test scores were not significant (all *p*’s > 0.07), yet the effect sizes for the pre-test scores indicated small-to-large differences (Cohen’s *d* = 0.11–0.81). Again, we do not find the pattern of results presented in Figure 2 as providing compelling evidence for the efficacy of videogame training to improve spatial abilities, for either men or women.

## Conclusion

The studies reviewed here do not represent all of the published evidence in support of the efficacy of videogame training to improve spatial abilities (see Powers et al., 2013, for review). However, the highlighted studies do illustrate the numerous challenges both scientists and laypersons face when trying to interpret the available research. In the three studies reviewed here, the sample sizes are smaller than the recommended minimum number of observations per cell for at least one of the comparison groups (Simmons et al., 2011), and the use of small samples leads to many problems. First, and most obvious, the use of small samples leaves the study underpowered to quantify the true effect of videogame training, and even more problematically leads to an increased likelihood of producing an inflated effect size (Button et al., 2013). Although meta-analyses are more informative than any individual study in quantifying the exact magnitude of the effect of videogame training, the meta-analysis is not a panacea if studies with small sample sizes produce large effect sizes that are then included in the meta-analysis. For example, Powers et al. (2013) provided Cohen’s *d* estimates of the effect sizes obtained in Maillot et al. (2012): *d* = 2.056 and *d* = 0.810 for matrix reasoning and directional headings, respectively. As reviewed above, the pattern of results for both the training and control groups were not straightforward, and inclusion of these large effect sizes in the meta-analysis will influence the overall meta-analytic estimate of the training effect size.

Second, although random sampling and assignment should eliminate pre-existing differences between the training and control groups, smaller samples provide less accurate estimates of the population values and will be more strongly influenced by an outlier value, and as such pre-test differences between training and control groups may be more likely. However, the use of small sample sizes also means that statistical tests of pre-test values are likely to be non-significant, allowing researchers to declare the training and control groups did not differ at pre-test (“*p* > 0.05”) despite the numerical differences between the groups. Note also that researchers are relying on failure to reject the null hypothesis as evidence for no difference between the training and control groups at pre-test (see Redick et al., 2013, for related discussion). As seen in the studies reviewed here (Figures 1 and 2), the control groups’ pre-test values were numerically but not significantly higher across several of the transfer tests, complicating interpretation of results when compared with the videogame training groups.

In closing, we offer a few suggestions for future videogame training studies. First, as noted in other recent reviews (Boot et al., 2011, 2013; Shipstead et al., 2012; Green et al., 2013), we strongly encourage the use of appropriate experimental control procedures that measure and counteract placebo effects. Second, for the reasons outlined above, we advocate the use of much larger sample sizes for training and control groups. Third, we strongly recommend presenting the pre- and post-test values for transfer tests, instead of or in addition to only reporting pre- to post-test change scores. Presenting the pre- and post-test values will allow the reader to determine if the pattern of significant results allows a strong conclusion or if there is ambiguity in the transfer results. A further suggestion is to provide the full data for each participant as supplemental material. Having the full data will be beneficial for interested readers and researchers conducting future meta-analyses, as they will be able to conduct both between- and within-subject analyses and additionally make comparisons not presented in the article by the authors. Finally, we note there is considerable debate about how best to statistically assess change in such intervention studies, with various authors pointing out limitations with independent-samples *t*-tests of gain scores, group by session interactions in ANOVA, and using pre-test scores as covariates in an ANCOVA (Lord, 1967; Huck and McLean, 1975; Miller and Chapman, 2001; Wright, 2006). Sampling techniques that lead to similar pre-test values for training and control groups will help minimize differences among the statistical analyses, whether that is achieved via random assignment with larger samples or through some sort of matching technique (for pros and cons of matching, see Green et al., 2013). In addition, incorporation of Bayesian analyses to either supplement or replace null-hypothesis significance-testing may more accurately quantify the intervention effect in a particular study (for a recent example of the use of Bayes Factors in cognitive training research, see Sprenger et al., 2013).

Above all, we hope that researchers will not focus so much on obtaining a significant *p*-value that they fail to examine the pattern of results to understand the cause of the significant result.

## Author contributions

Thomas S. Redick and Sean B. Webster contributed to the literature review. Sean B. Webster contacted authors for necessary additional information. Thomas S. Redick and Sean B. Webster created the figures, drafted the manuscript, and approved the final version for submission.

## Conflict of interest statement

The authors declare that the research was conducted in the absence of any commercial or financial relationships that could be construed as a potential conflict of interest.
